# Cancer cell repopulation after therapy: which is the mechanism?

**DOI:** 10.18632/oncoscience.577

**Published:** 2023-06-01

**Authors:** Rewati Prakash, Carlos M. Telleria

**Affiliations:** ^1^Experimental Pathology Unit, Department of Pathology, Faculty of Medicine and Health Sciences, McGill University, Montreal, QC, Canada; ^2^Cancer Research Program, Research Institute, McGill University Health Centre, Montreal, QC, Canada

**Keywords:** cancer cell repopulation, cancer stem cells, polyploidy, neosis, transitory senescence

## Abstract

Cancer cell repopulation after therapy is a phenomenon that leads to therapeutic failure with the consequent relapse of the disease. The process is understudied and mechanisms need to be uncovered. Here we discuss the issue of cancer cell repopulation after chemo- and radio-therapies. We compile evidence alleging that the repopulation of cancer cells can be originated from either cancer stem cells resistant to therapy, cancer cells that in response to therapy become polyploid and thereafter germinate into near-diploid rapid proliferating cells, and/or cells that respond to treatment undergoing senescence as a transient mechanism to survive, followed by the reinitiation of the cell cycle. Approaches targeted to prevent this post-therapy cancer cell repopulation should be uncovered to prevent tumor relapse and thus increase overall survival from this devastating disease.

## INTRODUCTION

**Figure 1:**
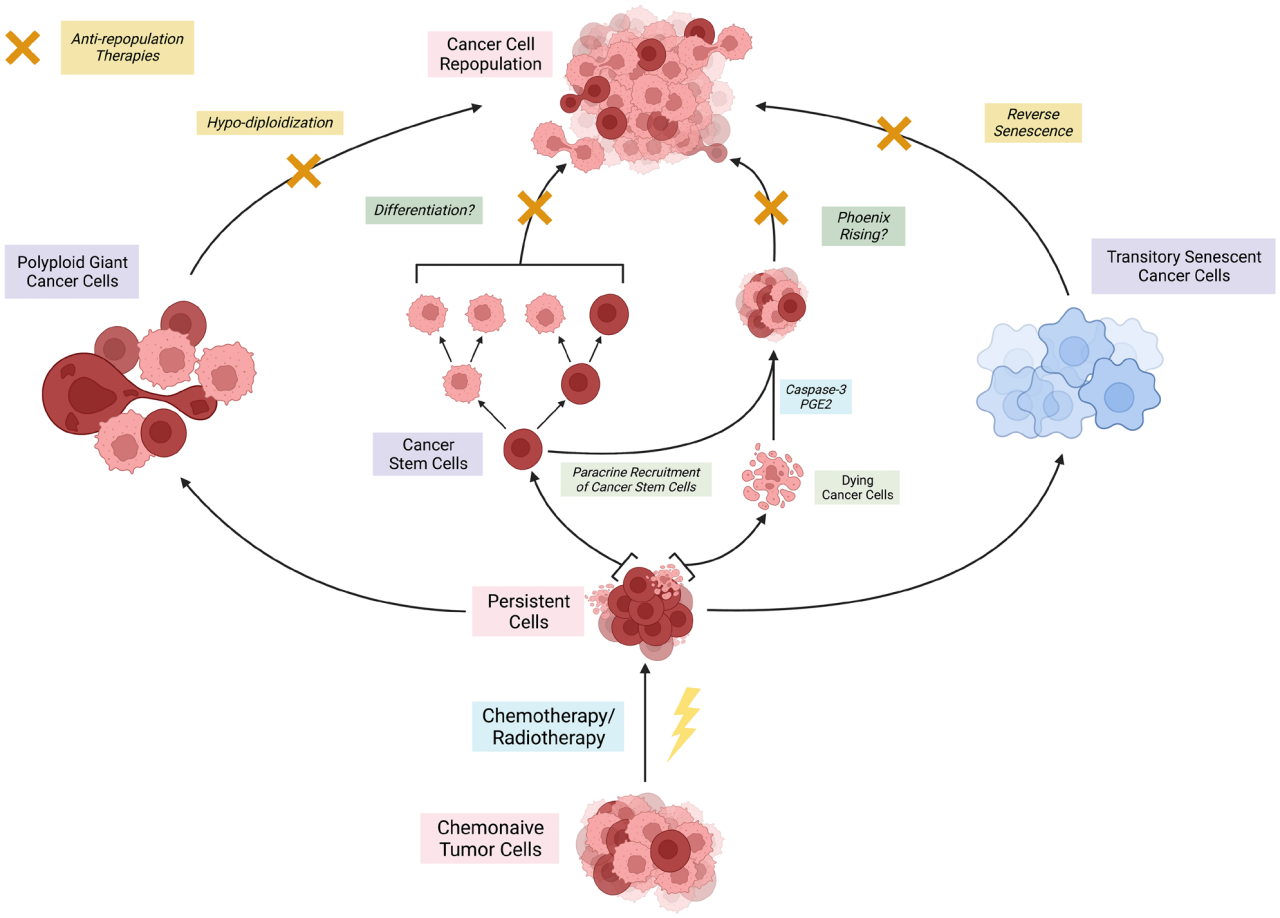
Presumed models of tumor cell repopulation after escaping chemoradiation.

The past two decades have brought great progress in the treatment of cancer as patients with the disease live longer having access to better diagnosis and therapeutic approaches. However, the disease remains incurable. One of the reasons for the high resilience of this disease is that cancer cells hide and escape from therapies thus leading to cancer recurrence. The process whereby cells escape therapy is referred to as cancer cell repopulation [1] which is a phenomenon that has been mathematically modeled [2]. It was first thought that this was a biological mechanism limited to the tumor microenvironment whereby chemo- and radio-therapies were not efficiently distributed within the tumor to kill all cells with the capacity to propagate the disease [3]. However, the phenomenon can be recreated *in vitro*. For instance, we have shown that ovarian and non-small cell lung cancer cells highly sensitive to platinum drugs repopulate a culture despite the fact that supra-pharmacological doses of the chemotherapeutic agents were utilized in the experiments [4–6]; even though the majority of the cells were killed by the treatments, always few cells remain in culture with the acquired capability to recreate a similar population of cancer cells if provided with nutrients, space, time, and not incurring in further insults. Likewise, cell repopulation has been demonstrated in breast and prostate cancers [7–9]. Nevertheless, despite being a well-documented phenomenon, the mechanisms involved in cancer cell repopulation remain poorly understood. In this perspective article we summarize three putative, not mutually exclusive molecular mechanisms that can drive the relapse of cancers via the regrowth of tumor cells that escape the initial treatment insult of chemotherapy and/or radiotherapy (Figure 1).

## PUTATIVE MECHANISMS

### Cancer stem cells

One of the first explanations for cancer cell repopulation is that the repopulating cells are derived from cancer stem cells. This theory implies that cancers are heterogeneous and have a progeny within which rapidly proliferating cells may be more easily killed by the therapy, sparing cells with stem-like phenotypes that are less differentiated and have cancer-initiating properties; they are termed cancer progenitor cells or cancer stem cells, which divide more slowly and therefore become spared by drugs and radiation. This residual therapy resistant population is capable to regenerate the disease via transit-amplifying cells originating from a niche enriched in cancer stem cells [10, 11]. These cells become abundant after therapy likely because of the hypoxic conditions of the tumor microenvironment [12]. Cancer stem cells reside in hypoxic niches and use energy from glycolysis to gain protection from reactive oxygen species (ROS) usually generated during oxidative phosphorylation; they also divide asymmetrically to preserve their identity by preventing further mutations [13]. To demonstrate their complexity, it has been shown that in many cancer types they can interconvert within the tumor niche into non-cancer initiating cells via the epithelial-to-mesenchymal transition [14]. In addition, they have unique biomarkers and signaling pathways that vary among cancer types [15, 16]; these markers and pathways are essential to locate these scarce cells within a tumor. Whereas standard chemo- and radio-therapies seem to kill more differentiated cancer cells than cancer initiating cells [17], the ones with cancer stem cell properties develop resistance to treatment, persist within a niche, and regenerate a tumor by induction of proliferation upon escape from mitotic arrest [17].

It has been reported that the stem-like cells that resist treatment may also be influenced in a paracrine manner by dying cancer cells through a mechanism initially reported in wound healing regenerative processes called “Phoenix rising”; this program involves cell death followed by compensatory proliferation driven by active caspase-3 leading to the generation of arachidonic acid as precursor of prostaglandin E2, which is needed for stem cell proliferation [9, 17, 18]. This phenomenon has been mathematically modeled [19] and is a putative mechanism whereby therapy-induced dying cells release factors that promote cancer cell repopulation.

A long-term yet challenging approach to abrogate the repopulating capacity of a tumor requires targeted approaches, which may not affect the bulk of the tumor, yet may eradicate the cancer propagating capacity of cancer stem cells only; targeting the slow dividing cancer initiating cells should lead to impaired recurrence [13, 16]. Finally, targeting pathways within the cancer stem cell niche or using immunotherapeutic strategies may provide a good opportunity to prevent cancer cell repopulation [20–22].

### Polyploid giant cancer cells

Another feasible mechanism to justify cancer cell repopulation is that repopulating cells are derivatives of polyploid giant cancer cells formed in response to therapies. These giant cells can be induced by hypoxia, chemotherapy, and radiation therapy [23–26]. However, with time, the polyploid giant cells are capable to give rise to smaller, near diploid highly proliferative cells via a mechanism coined as “neosis”, “hypo-diploidization”, or “reverse ploidy” [27, 28]. This is a modality of cell division generated in cells that escape mitotic crisis by undergoing budding followed by nuclear division and asymmetric cytokinesis resulting in the formation of aneuploid and mitotically active cells with genome stability [29, 30]. The polyploid giant cells are usually formed in response to the stress of chemoradiation, with the majority of them undergoing cell death. However, few survive isolating the chromosomes required by diploid tumor cells and producing a chemoresistant progeny via depolyploidization [31–33]. The polyploid giant cells arise as a consequence of endoreplication leading to the formation of mononucleated giant cells first, followed by cytokinetic failure and cell-cell fusion, thus giving rise to daughter cells able to proliferate for a long-term and carrying new chromosome alterations that cause distant metastases [32, 34, 35]. The emerging cells from polyploidy giant progenitors inherit stem-like properties [36] with capacity to differentiate into multiple malignant cell types [37]. The polyploid giant cells give rise to a near diploid progeny that gradually develops a more aggressive phenotype with passaging [38]. The overall process of hypo-diploidization utilizes meiosis-specific genes while cells eliminate excess chromatin [39, 40]. It is anticipated that uncovering the mechanism of reverse ploidy should guide researchers into developing targeted therapies that may prevent tumor recurrence.

### Transient senescence cells

Growing evidence supports the idea that cancer cell repopulation happens as a consequence of transitory senescence. It is hypothesized that a rare percentage of cancer cells escape treatment by undergoing transient cell cycle arrest acquiring a senescence phenotype. This phenomenon has been often considered irreversible and characterized by cells having a flat morphology, expressing senescence-associated beta galactosidase due to enlargement of the lysosomal compartment, with formation of heterochromatic foci and endowed with a unique secretory program [41, 42]. One of the most important function of senescence is tumor suppression as these cells limit tumor progression by upregulating p53, p16, and p21, and are cleared by the immune system to limit tumorigenesis in usually premalignant lesions, or following cancer therapy [43, 44]. However, if senescence persists, it can also have detrimental effects in cancer tissues because the secretory phenotype of senescence cells generate a pro-inflammatory condition that favors tumor progression [42, 45].

The irreversibility of the senescence phenotype has nonetheless been heavily disputed. For instance senescent cells with low expression of tumor suppressor p16^ink4^ resume cell growth upon inactivation of tumor suppressor p53 [46], p53 null lung cancer cells escape senescence induced by various drugs including cisplatin, camptothecin, etoposide, paclitaxel and vindesine by upregulating cyclin-dependent kinase 1 (Cdk1) [47], senescent colon and breast cancer cells regain proliferative capacity upon exposure to doxorubicin [48, 49], whereas senescent melanoma cells proliferate again upon the overexpression of the inhibitor of apoptosis protein survivin [50]. Escape from senescence has been also reported in breast cancer cell cultures exposed to conventional chemotherapy with the escaping cells expressing stem cell markers (high CD133 and Oct-4), low levels of ROS, and increased antioxidant enzymes [51]. Reinforcing the concept that indeed a senescence phenotype is not irreversible, a recent work, using a model of acute myeloid leukemia (AML) demonstrates that following chemotherapy, AML cells enter a senescence-like phenotype that repopulate the tumor leading to AML recurrence [52]. Of interest the phenomenon occurred by induction of embryonic diapause-like dormancy transcriptional signature and stemness reprogramming. It was also shown in this model that the induction of senescence, tumor survival and tumor persistence is dependent on ataxia telangiectasia and Rad3-related protein (ATR) involved in DNA damage/repair. In sum, there is mounting evidence that senescence is a transitional mechanism that can be induced by an array of therapies in various cancers and that is reversible with cells re-entering the cell cycle and repopulating a tumor. Consequently, it is questionable whether developing drugs to induce senescence as a manner to arrest cell growth in tumors is a clever way to stop cancer recurrence. Instead, usage of senolytic agents such as anti-BCL-2 family of proteins [42] or ATR inhibitors [52] may be a manner to eliminate transitory senescence cells. Perhaps the better approach to eliminate cancer cell repopulation is a combination treatment involving first chemoradiation-induced transitory senescence, followed by senolytic therapies as recently discussed by Wang and colleagues [42].
